# Physiologically based kinetic modeling of the bioactivation of myristicin

**DOI:** 10.1007/s00204-016-1752-5

**Published:** 2016-06-22

**Authors:** Amer J. Al-Malahmeh, Abdelmajeed Al-Ajlouni, Sebastiaan Wesseling, Ans E. M. F. Soffers, Ala’ Al-Subeihi, Reiko Kiwamoto, Jacques Vervoort, Ivonne M. C. M. Rietjens

**Affiliations:** 1 Division of Toxicology, Wageningen University, Building 124, Stippeneng 4, 6708 WE Wageningen, The Netherlands; 2 Aqaba International Laboratories/BENHAYYAN, ASEZA, Aqaba, 77110 Jordan; 3 Department of Biochemistry, Wageningen University, Building 124, Stippeneng 4, 6708 WE Wageningen, The Netherlands; 4 Faculty of Pharmacy, Philadelphia University, P.O. Box 1, Amman, 19392 Jordan

**Keywords:** Myristicin, Alkenylbenzenes, Safrole, Physiologically based kinetic (PBK) modeling, Read-across-based risk assessment

## Abstract

The present study describes physiologically based kinetic (PBK) models for the alkenylbenzene myristicin that were developed by extension of the PBK models for the structurally related alkenylbenzene safrole in rat and human. The newly developed myristicin models revealed that the formation of the proximate carcinogenic metabolite 1′-hydroxymyristicin in liver is at most 1.8 fold higher in rat than in human and limited for the ultimate carcinogenic metabolite 1′-sulfoxymyristicin to (2.8–4.0)-fold higher in human. In addition, a comparison was made between the relative importance of bioactivation for myristicin and safrole. Model predictions indicate that for these related compounds, the formation of the 1′-sulfoxy metabolites in rat and human liver is comparable with a difference of <2.2-fold over a wide dose range. The results from this PBK analysis support that risk assessment of myristicin may be based on the BMDL_10_ derived for safrole of 1.9–5.1 mg/kg bw per day. Using an estimated daily intake of myristicin of 0.0019 mg/kg bw per day resulting from the use of herbs and spices, this results in MOE values for myristicin that amount to 1000–2700, indicating a priority for risk management. The results obtained illustrate that PBK modeling provides insight into possible species differences in the metabolic activation of myristicin. Moreover, they provide an example of how PBK modeling can facilitate a read-across in risk assessment from a compound for which in vivo toxicity studies are available to a related compound for which tumor data are not reported, thus contributing to alternatives in animal testing.

**Electronic supplementary material:**

The online version of this article (doi:10.1007/s00204-016-1752-5) contains supplementary material, which is available to authorized users.

## Introduction

**Fig. 1:**
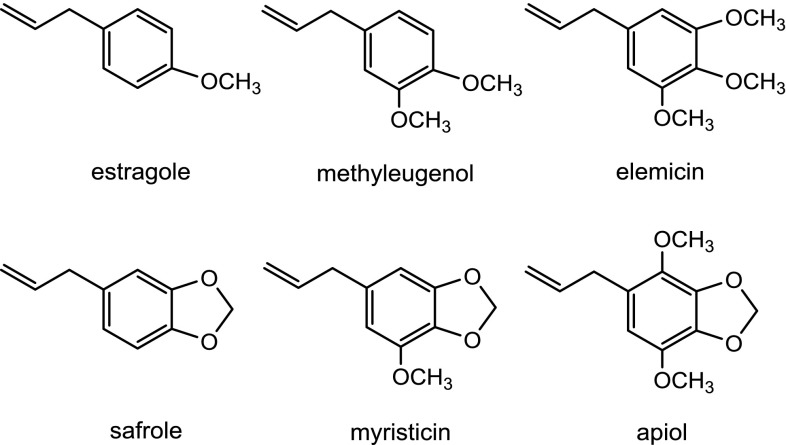
Structural formulas of the alkenylbenzenes, estragole, methyleugenol, elemicin, safrole, myristicin, and apiol

Myristicin (1-allyl-5-methoxy-3,4 methylene-dioxybenzene or methoxysafrole) is naturally occurring and present in several spices including nutmeg and mace of trees of Myristica species, principally Myristica fragrans Hout, and their essential oils (Forrest and Heacock 1972; Matthews et al. 1974; Sammy and Nawar 1968). There is a potential for human exposure to myristicin through foods, beverages, food supplements, and traditional medicines. Myristicin belongs to the group of alkenylbenzenes that contains structural analogues such as methyleugenol, estragole, elemicin, safrole, and apiol (Fig. 1), compounds that are all naturally occurring in herbs and spices such as basil, nutmeg, and their essential oils (Barceloux 2009). Safrole, estragole, and methyleugenol have been shown to induce hepatic tumors in rats or mice upon chronic oral exposure to high doses and upon administration to male CD-1 mice during the preweaning period (Borchert et al. 1973; Drinkwater et al. 1976; Ioannides et al. 1981; Wislocki et al. 1977; Innes 1969; National Toxicology 2000). Tumors were found especially in the liver at frequencies that amounted to, for example, 2/50, 3/50, 14/50, and 25/50 at 0, 37, 75, and 150 mg methyleugenol/kg bw per day in male rats (National Toxicology 2000). A summary of further incidences of malignant tumor formation in mice and rats after administration of estragole, methyleugenol, or safrole can be found in our previous paper (van den Berg et al. 2011). However, myristicin is less well studied than its structurally related analogues, and only limited toxicological data are available. While in vitro genotoxicity studies indicate that myristicin is mutagenic and capable of inducing the formation of DNA adducts (EFSA 2012; Zhou et al. 2007b), no two-year carcinogenicity studies of myristicin in experimental animals are available hampering its risk assessment.

Some short-term studies were conducted on the induction of hepatic tumors, in which male B6C3F1 mice were given myristicin during the preweaning period in two separate experiments. In the first experiment, myristicin was injected with a total dose of 3.75 µmol to 33 male B6C3F1 mice over a period of 22 days, and the total duration of the experiment was 12 months. In a second experiment, 45 male B6C3F1 mice were each injected with a total dose of 4.75 µmol myristicin over a period of 22 days, and the total duration of the experiment was 18 months. In these short-term exposure experiments, myristicin had no detectable activity for the initiation of hepatic tumors (Miller et al. 1983).

**Fig. 2:**
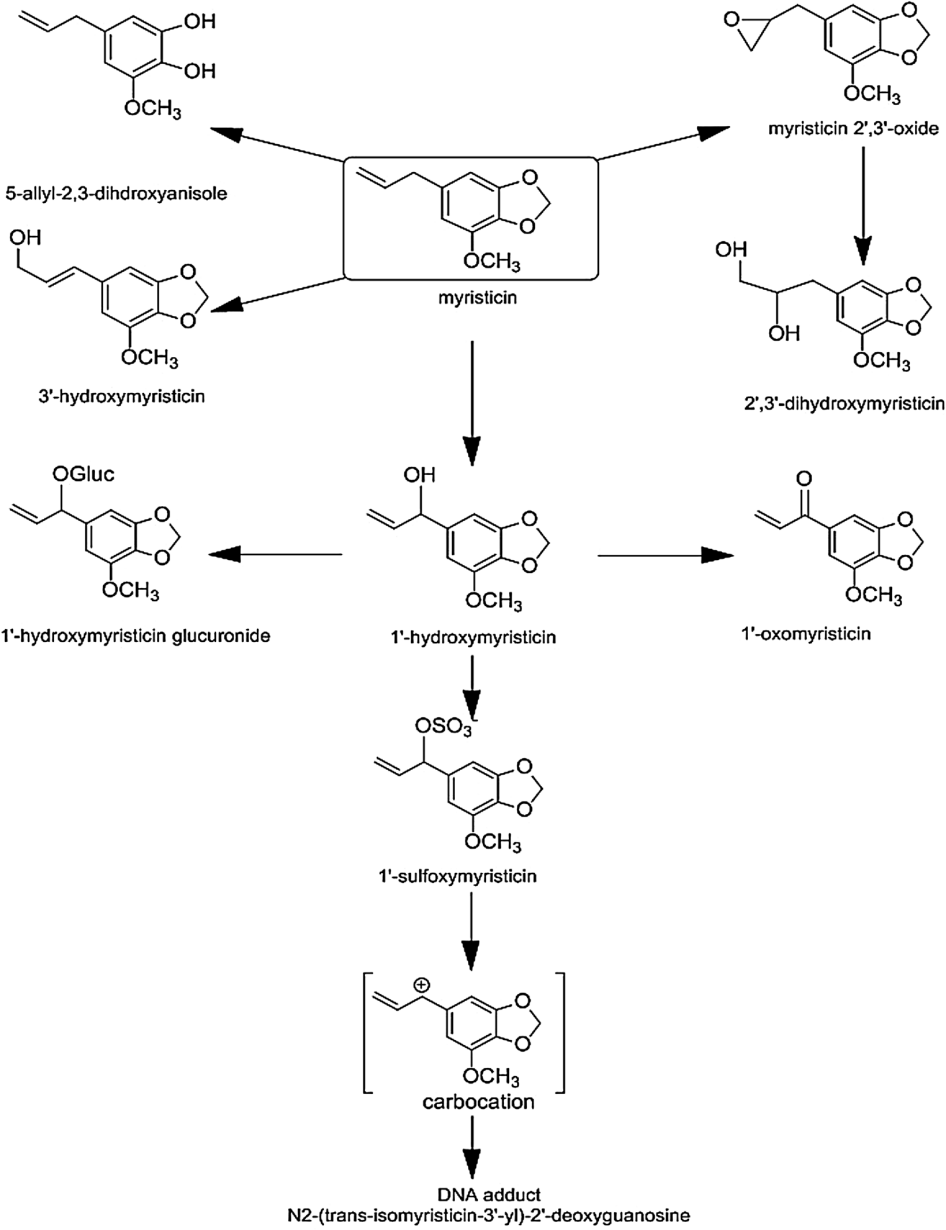
Proposed metabolic pathways of the alkenylbenzene myristicin

 In agreement with hepatocarcinogenicity in mice (Miller et al. 1983), DNA adduct formation upon exposure to myristicin was generally lower and less persistent than DNA adduct formation upon exposure to methyleugenol and estragole and appeared to be about twofold lower than DNA adduct formation upon exposure to safrole, both in in vitro study (Zhou et al. 2007a) and in in vivo studies (Table 1) (Phillips et al. 1984; Randerath et al. 1984). N^2^-(trans-isomyristicin-3′-yl)-2′-deoxyguanosine was found to be the major myristicin DNA adduct formed when mice were given cola drinks instead of water up to 8 weeks. In a parallel experiment, pregnant ICR mice were treated by gastric intubation with a single dose of 6 mg of myristicin, and the level of myristicin adducts in maternal and fetal liver was 68 and 63 % of the total adducts (Randerath et al. 1993). In freshly isolated rat hepatocytes in primary culture, safrole, estragole, and methyleugenol induced unscheduled DNA synthesis **(**UDS) and cytotoxicity at the concentrations of 10^−6^ to 10^−3^ M, (Howes et al. 1990) while myristicin showed cytotoxicity. In these studies, concentrations inducing UDS were generally close to or already at concentrations detecting cytotoxicity, hampering interpretation of the data. The data supporting a genotoxic mode of action for the tumor induction by the alkenylbenzenes rather come from studies reporting DNA adduct formation. In these studies, myristicin, as well as estragole, methyleugenol, and safrole, all showed positive results (Zhou et al. 2007a; Kobets et al. 2016; Phillips et al. 1984; Randerath et al. 1984). A schematic overview of the detoxification and bioactivation of myristicin that is similar to that of its structurally related compound safrole (Swanson et al. 1979; Borchert et al. 1973; Drinkwater et al. 1976) is shown in Fig. 2. Epoxidation of the double bond in the allyl side chain yields the 2,3-epoxide. In in vitro experiments, the epoxide readily forms DNA adducts, but rapid detoxification by epoxide hydrolase and glutathione *S*-transferases (GSTs) prevents it from forming detectable levels of DNA adducts in vivo (Luo et al. 1992; Luo and Guenthner 1996). The primary bioactivation pathway of myristicin is 1′-hydroxylation of the alkene side chain to yield the 1′-hydroxy metabolite which can be conjugated with either glucuronic acid representing a detoxification reaction or sulfate representing the ultimate bioactivation to 1′-sulfoxymyristicin (Drinkwater et al. 1976; Benedetti et al. 1977; Zangouras et al. 1981; Miller et al. 1983). The 1′-sulfoxy metabolite represents the ultimate carcinogenic metabolite (Wiseman et al. 1985, 1987; Randerath et al. 1984; Phillips et al. 1984). 1′-Sulfoxy metabolites of alkenylbenzenes may react readily with DNA, RNA, and proteins but can also be detoxified through reaction with H_2_O or conjugation with glutathione (Phillips et al. 1984; Miller et al. 1983; Fennell et al. 1984; Ishii et al. 2011). Therefore, only a fraction of the 1′-sulfoxy metabolite is expected to form DNA adducts (Rietjens et al. 2014).

**Table 1:** Comparison of data on DNA adduct formation from in vitro and rodent studies using safrole and myristicin

Model	Sex	Route of admin.	Myristicin		Safrole		DNA adducts identified	Duration of exposure	DNA isolation and persistence study	Analysis technique of DNA adduct	Refs.
			Dose or conc.	pmol Adduct/mg DNA	Dose or conc.	pmol Adduct/mg DNA					
Cultured human HepG2 cells			50^#^	0.017	50^#^		N2-(trans-propenylbenzene-3′-yl) deoxyguanosine (major) and N2-(allylbenzene-1′-yl) deoxyguanosine (minor)	The cells were treated with different doses, incubated, and then harvested after 24 h		^32^P-Post-labeling, studying DNA binding ability in vitro	Zhou et al. (2007a)
			150^#^	0.049	150^#^	0.083					
			450^#^	0.165	450^#^	0.102					
Mouse liver CD-1	Female	i.p	400^*^	49.5	400^*^	203.4	N2-(trans-propenylbenzene-3′-yl) deoxyguanosine and N2-(allylbenzene-1′-yl) deoxyguanosine (both major), N6–(trans -propenylbenzene-3′-yl) deoxyadenosine (minor)	Livers were collected 24 h after treatment	After admin. of dose 400 mg/kg bw safrole, livers were taken after 24 h, 28, 58, 98, and 140 days after each treatment, after 140 days adduct was 21 pmol/mg DNA	^32^P-Post-labeling, studying DNA binding ability in vivo	Randerath et al. (1984)
			80^*^	16.8	80^*^	45.9					
New born mice B6C3F1	Male	i.p	36.5^*^	7.8	30.8^*^	17.5	N2-(trans & cis-propenylbenzene-3′-yl) deoxyguanosine and N2-(allylbenzene-1′-yl) deoxyguanosine (both major) (both major) N6–(trans -propenylbenzene-3′-yl) deoxyadenosine (minor)	22 days after birth (1, 8, 15, and 22 days of injection)	Days 23, 29, and 43. adducts were still available after 43 days	^32^P-Post-labeling, studying DNA binding ability in vivo	Phillips et al. (1984)

^#^For concentration in uM

^*^For dose in mg/kg bw

Although myristicin is thus likely to also induce DNA adducts and liver tumors, its risk assessment at low realistic dietary intake levels is hampered by the fact that carcinogenicity studies are lacking (Hallstrom and Thuvander 1997). Therefore, the aim of the present study was to characterize the detoxification and bioactivation of myristicin and to develop a physiologically based kinetic (PBK) model to describe the ultimate formation of the 1′-sulfoxy metabolite in the liver of both rat and human and subsequently perform a read-across-based risk assessment using a similar PBK model previously described for safrole for which tumor data from long-term toxicity testing are available. Previously, using a similar approach and read-across from estragole and methyleugenol, the risk of exposure to elemicin for which also only limited in vivo rodent tumor data were available could be evaluated (van den Berg et al. 2012). Such a PBK modeling-based read-across illustrates a novel approach for animal-free risk assessment of a genotoxic carcinogen without the need for a long-term carcinogenicity study.

## Materials and methods

### Chemicals

Myristicin, tris(hydroxymethyl)aminomethane, uridine 5′-diphosphoglucuronide acid (UDPGA), reduced L-glutathione (GSH), alamethicin (from Trichodermaviride), 3′-phosphoadenosine-5′-phosphosulfate (PAPS), β-nicotinamide adenine dinucleotide (NAD^+^), and reduced β-nicotinamide adenine dinucleotide phosphate (NADPH) were obtained from Roche Diagnostics (Mannheim, Germany). Dimethyl sulfoxide (DMSO) was obtained from Acros Organics (Geel, Belgium). Potassium dihydrogen phosphate, dipotassium hydrogen phosphate trihydrate, acetic acid, and magnesium chloride were supplied by VWR International (Darmstadt, Germany). Acetonitrile (ACN) (UPLC/MS grade) was purchased from Biosolve BV (Valkenswaard, The Netherlands). Trifluoroacetic acid TFA was obtained from Merck (Darmstadt, Germany). Pooled male rat liver microsomes and S9 from Sprague–Dawley and mixed gender pooled human liver microsomes and S9 were obtained from BD Gentest (Woburn, USA). Pooled male Sprague–Dawley rat lung, kidney, and small intestinal microsomes and pooled gender human lung, kidney, and intestinal microsomes were purchased from BioPredic International (Rennes, France).

### Synthesis of 1′-hydroxymyristicin and 1′-oxomyristicin

The synthesis of 1′-hydroxymyristicin from myristicin was done as described previously by Jeurissen et al. (2004), and 1′-oxomyristicin was synthesized from 1′-hydroxymyristicin according to the method used for the synthesis of 1′-oxoestragole from 1′-hydroxyestragole (Wislocki et al. 1976).

### Microsomal metabolism of myristicin

First, it was determined which organs are involved in the biotransformation of myristicin in rat and human. For this purpose, liver, kidney, lung, and small intestine microsomes from male Sprague–Dawley rat and pooled gender human were used. Incubations were performed by adding myristicin to incubation mixtures containing 1 mg/mL of the microsomal protein preparations and 3 mM NADPH in 0.2 M Tris–HCl (pH 7.4). After 1 min of pre-incubation at 37 °C, myristicin (final concentration, 1000 μM) was added from a 100 times concentrated stock solution in DMSO so that the final DMSO content was 1 % (v/v). Incubations were carried out for 30 min after which the reactions were terminated by adding 25 µl (0.25 times the incubation volume) of ice-cold ACN. Samples were centrifuged for 5 min at 13,000×*g*, and the supernatant was stored at −20 °C until UPLC analysis. As metabolism of myristicin was observed only in incubations with liver microsomes for male rats and pooled gender human, the determination of kinetic constants for the formation of microsomal metabolites was performed only for the liver fractions. Incubations to determine kinetic constants were performed following the conditions described above using final concentrations of myristicin from 25 to 1000 μM for rat and human liver. In all incubations, the final concentration of DMSO, in which myristicin was dissolved, was kept at 1 % (v/v). The formation of different microsomal metabolites was linear with time and microsomal protein concentration under the conditions described. Blank incubations were performed in the absence of the cofactor NADPH. All incubations were performed in triplicate.

### Glucuronidation of 1′-hydroxymyristicin to 1′-hydroxymyristicin glucuronide

The kinetic constants for the metabolic conversion of 1′-hydroxymyristicin to 1′-hydroxymyristicin glucuronide (HMG) by both male rat and human liver fractions were determined as described previously for the related 1′-hydroxyalkenylbenzenes (Punt et al. 2008; van den Berg et al. 2012; Punt et al. 2009; Martati et al. 2011, 2012; Al-Subeihi et al. 2011, 2012). Briefly, incubations contained (final concentrations) 10 mM of UDPGA and 0.5 mg/ml of male Sprague–Dawley or pooled gender human S9 protein in 0.2 M Tris–HCl (pH 7.4) containing 10 mM of MgCl_2_. To overcome enzyme latency and to obtain maximal glucuronidation activity, incubations containing S9 were pretreated on ice with 0.025 mg/ml alamethicin added from a 200 times concentrated stock dissolved in methanol (Fisher et al. 2000; Lin and Wong 2002). After 15 min of alamethicin treatment, samples were pre-incubated at 37 °C for 1 min, and reactions were subsequently started by adding 1′-hydroxymyristicin at final concentrations of 10 to 1200 μM. 1′-Hydroxymyristicin was added from 100 times concentrated stock solutions in DMSO. The reaction mixtures were incubated for 30 and 180 min for rat and human, respectively, and terminated by adding 25 µl (0.25 times the incubation volume) of ice-cold ACN. Blank incubations were carried out in the absence of the cofactor UDPGA. Experiments were performed in triplicate. The longer incubation time in human samples was required due to the lower rate of glucuronidation. The formation of 1′-hydroxymyristicin glucuronide was linear with time and the S9 protein concentration under the experimental conditions described. All samples were centrifuged for 5 min at 16,000×*g*, and the supernatant was stored at −20 °C until ultra-performance liquid chromatography (UPLC) analysis.

### Oxidation of 1′-hydroxymyristicin to 1′-oxomyristicin

The kinetic constants for the enzymatic conversion of 1′-hydroxymyristicin to 1′-oxomyristicin were determined using incubation mixtures containing (final concentrations) 3 mM NAD^+^, 2 mM GSH, and 1 mg/ml rat or human liver microsomes in 0.2 M Tris–HCl (pH 7.4). GSH was added to the incubation mixtures to trap the reactive 1′-oxo metabolite formed after oxidation of 1′-hydroxymyristicin. The level of GSH in the incubations was optimized in a previous study to obtain maximum scavenging of 1′-oxoestragole (Punt et al. 2009). To this end, incubations were performed in the presence of increasing concentrations of GSH, ranging from 2 to 10 mM. At a concentration of 2 mM GSH maximum formation of GS-1′-oxoestragole was reached in the incubations, pointing at maximum scavenging of 1′-oxoestragole at this concentration (Punt et al. 2009). Kinetic constants for this reaction in rat and human liver were derived by performing incubations with NAD^+^ as a cofactor, given that in rat and human liver, NAD^+^ is mainly present in an oxidized form with levels of NAD^+^ being much higher than those of NADH. In a previous study, it was shown that the highest level of 1′-hydroxy alkenylbenzene oxidation occurs in incubations with microsomes in the presence of NAD^+^ as cofactor. In incubations with pooled human liver cytosol in the presence of NAD^+^ or NADP^+^, lower levels of oxidation were observed, indicating that the reaction is not primarily catalyzed by alcohol dehydrogenases (ADH) or other enzymes present in the cytosol (Punt et al. 2010). The enzyme responsible for the oxidation may be 17β-hydroxysteroid dehydrogenase type 2 (17β-HSD2) (Punt et al. 2010). Prior to the addition of 1′-hydroxymyristicin at final concentrations ranging between 10 and 4000 μM to the incubation mixture from 100 times concentrated stock solutions in DMSO, samples were pre-incubated for 1 min at 37 °C. Reactions were terminated after 10 min of incubation at 37 °C by adding 25 μl (0.25 times the incubation volume) of ice-cold ACN. The formation of the GSH conjugate of 1′-oxomyristicin (GS-1′-oxomyristicin) was linear with time and microsomal protein concentration under the experimental conditions used. Blank incubations were performed without the cofactor NAD^+^. Incubations were performed in triplicate. All samples were centrifuged for 5 min at 13,000×*g*, and the supernatant was stored at −20 °C until UPLC analysis.

### Sulfonation of 1′-hydroxymyristicin to 1′-sulfoxymyristicin

The formation of 1′-sulfoxymyristicin was determined using incubations containing male rat liver Sprague–Dawley or pooled gender human liver S9 proteins, PAPS as a cofactor, and GSH, which acts as a scavenger of the reactive carbocation formed due to the unstable nature of the 1′-sulfoxy metabolite in an aqueous environment (van den Berg et al. 2012; Martati et al. 2011; Al-Subeihi et al. 2011). Incubation mixtures containing (final concentrations) 10 mM of GSH, 0.2 mM of PAPS, and 3 mg/ml of S9 proteins in 0.1 M potassium phosphate (pH 8.2) were pre-incubated for 1 min at 37 °C. After the pre-incubation, 1′-hydroxymyristicin dissolved in DMSO was added in final concentrations ranging between 10 and 6000 μM while keeping the final content of DMSO at 1 % (v/v). The reaction was terminated after 360 min of incubation by adding 25 μl (0.25 times the incubation volume)  of ice-cold ACN. The formation of the GSH conjugate of 1′-sulfoxymyristicin was linear with time and S9 protein concentrations under the experimental conditions used. Blank incubations were performed in the absence of PAPS. Incubations were performed in triplicate. All samples were centrifuged for 5 min at 16,000×*g*, and the supernatant was stored at −20 °C until ultra-performance liquid chromatography (UPLC) analysis.

### Identification and quantification of metabolism of myristicin and 1′-hydroxymyristicin by UPLC

Microsomal incubations with myristicin only detected primary metabolites, and all incubation conditions were chosen such that substrate conversion did not exceed 10 % of the initial substrate concentration, a condition that is also essential to ascertain adequate determination of the kinetic constants. Secondary metabolism of the relevant 1′-hydroxy metabolite of myristicin was taken into account by determining the kinetic constants for its conversion in (a) glucuronidation, (b) sulfation, and (c) oxidation, again under conditions that allowed adequate definition of kinetic constants and with <10 % substrate conversion. Incubation samples were subjected to UPLC analysis that was performed as described previously (Punt et al. 2008). Identification was achieved by comparing the UV spectra of the formed metabolites to the spectra of the synthesized 1′-hydroxymyristicin and 1′-oxomyristicin reference standards. Quantification of all formed metabolites was done by comparing the peak areas to those of calibration curves of the corresponding reference compounds, determined using UPLC with photodiode array detection (UPLC-PDA). The UPLC system was composed of a Waters (Waters, Milford, MA) Acquity solvent manager, sample manager, and photodiode array detector, equipped with Water Acquity UPLC BEH C18 column.

For 2′,3′-dihydroxymyristicin and 5-allyl-2,3-dihydroxyanisole that were found to have the same UV spectrum as 1′-hydroxymyristicin, and for 1′-hydroxymyristicin, estimation was based on the comparison of the peak area of the formed metabolites to the calibration curve of the synthesized 1′-hydroxymyristicin at a wavelength of 210 nm. For 3′-hydroxymyristicin, estimation was based on the comparison of the peak area to the calibration curve of the GSH adduct of synthetised 1′-oxymyristicin at a wavelength of 212 nm, because 3′-hydroxymyristicin was found to have the same UV spectrum as the GSH adduct of synthetised 1′-oxymyristicin. The gradient for analysis of microsomal metabolites was performed with 100 % ACN and ultrapure water containing 0.1 % (v/v) TFA. The flow rate was 0.6 ml/min. The gradient started at 10 % ACN, increased to 50 % ACN over 4 min, increased to 80 % over 0.5 min, followed by decrease to 10 % ACN over 0.5 min, and finally kept at 10 % for 1 min.

1′-Hydroxymyristicin glucuronide was estimated based on the comparison of the peak area of the formed metabolite to the calibration curve of 1′-hydroxymyristicin at a wavelength of 210 nm. The flow rate was 0.6 ml/min. The gradient was made using a mixture of ACN and ultrapure water containing 0.1 % (v/v) TFA. The gradient started at 10 % ACN, increased to 60 % over 3.5 min, after which ACN was increased to 80 % over 0.5 min, and kept at 80 % for 0.5 min, and finally decreased to 10 % over 0.5 min.

Quantification of GS-1′-oxomyristicin was based on a calibration curve of the GSH adduct of the synthesized 1′-oxymyristicin made as previously described (van den Berg et al. 2012; Punt et al. 2009; Martati et al. 2011; Al-Subeihi et al. 2011). In short, a 60 μM concentration of the synthetic standard of 1′-oxomyristicin, dissolved in ACN, was incubated with different concentrations of GSH (i.e., 0–20 μM) in 0.2 M Tris–HCl (pH 7.4) for 6 h at 37 °C, resulting in full conversion of the GSH to GS-1′-oxymyristicin. Quantification was done by comparing the peak areas of the formed GS-1′-oxo metabolite in the incubation mixtures with peak areas of the GS-1′-oxymyristicin calibration curve thus obtained at a wavelength of 212 nm. The gradient for analysis of the metabolite consisted of a mixture of ACN and ultrapure water containing  0.1 % (v/v) TFA. The flow rate was 0.6 ml/min, The gradient started with 10 % ACN, increased to 30 % ACN over 2.5 min, after which ACN was increased to 80 % over 0.5 min, kept at 80 % for 0.5 min, followed by a decrease to 10 % over 0.5 min, and finally kept at 10 % for 0.5 min.

Quantification of 1′-sulfoxymyristicin was done using UPLC analysis as described for the estimation of 1′-sulfoxysafrole (Martati et al. 2011). The UV spectrum of the GSH adduct of 1′-sulfoxymyristicin was found to be similar to the UV spectrum of the GSH conjugate of 1′-oxomyristicin, and estimation of the GSH adduct of 1′-sulfoxymyristicin was thus accomplished by comparing the peak area of this metabolite to the calibration curve of GS-1′-oxomyristicin at a wavelength 305 nm. The gradient for analysis of the metabolite consisted of a mixture of ACN and ultrapure water containing 0.1 % (v/v) TFA. The flow rate was 0.6 ml/min, starting at 0 % ACN and increasing the percentage of ACN to 20 % over 0.2 min, followed by an increase to 30 % ACN over 4.3 min, then increasing to 100 % over 0.3 min, and keeping it at 100 % for 0.2 min, and finally decreasing to 0 % over 0.2 min and keeping it at 0 % for 0.8 min. Separation and purification of the GSH adduct of 1′-sulfoxymyristicin was performed by collecting the peak of the metabolite from the UPLC column. Then, LC–MS analysis of the metabolite was conducted using a micro-TOF MS (Bruker) coupled to an Agilent LC (1200 Series) equipped with Altima C18 column (150 × 4.6 mm, 3 µm). The mobile phase used consisted of (A) nanopure water with 0.1 % formic acid and (B) HPLC-grade ACN with 0.1 % formic acid. Elution was at a flow rate of 0.8 ml/min, starting at 22 % B with a linear increase to 100 % B in 30 min. Subsequently, the gradient returned linearly to the initial conditions in 2 min and remained 13 min at this condition prior to the next injection. Mass spectrometric analysis was in the negative electrospray mode using a spray capillary voltage of 4500 V, a capillary temperature of 200 °C, and nitrogen as nebulizer gas at 8.0 L/min.

### Determination of kinetic constants

Kinetic constants for the metabolic conversions of myristicin and 1′-hydroxymyristicin were derived by fitting the data to the standard Michaelis–Menten equation,

$$v = V_{ \hbox{max} } \times \left[ S \right]/K_{\text{m}} + \left[ S \right]$$

For conversion of 1′-hydroxymyristicin to 1′-sulfoxymyristicin, a first-order rate linear equation was used:

$$v = k_{\text{HMS}} \times \left[ S \right]$$

in which [*S*] represents the substrate concentration, *V*_max_ the maximum velocity, and *K*_m_ the Michaelis–Menten constant for the formation of the different metabolites of myristicin or 1′-hydroxymyristicin, *k*_HMS_ the first-order rate constant for sulfonation of 1′-hydroxymyristicin. Data analysis was accomplished using GraphPad Prism, version 5.04 (GraphPad Software, San Diego, CA).

### Physiologically based kinetic (PBK) models

**Fig. 3:**
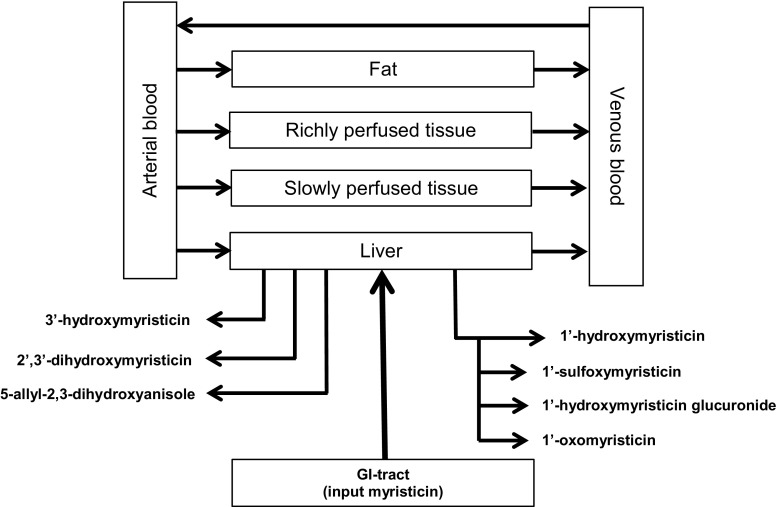
Schematic overview of the PBK model for myristicin in rat and human

Two PBK models were developed describing the relative importance of bioactivation and detoxification of myristicin in rat and human at different oral dose levels. The models developed in this study were essentially based on the PBK models previously defined for the metabolism of estragole (Punt et al. 2008, 2009), methyleugenol (Al-Subeihi et al. 2011, 2012), elemicin (van den Berg et al. 2012), and safrole (Martati et al. 2011, 2012) in rat and human. A schematic overview of the developed PBK models for myristicin metabolism in rat and human is shown in Fig. 3. The models consist of several compartments representing different organs and tissues (i.e., liver, fat tissue, richly perfused tissues, and slowly perfused tissues) that are mutually connected through the systemic circulation. First-order kinetics was used to describe the uptake of myristicin from the gastrointestinal (GI) tract assuming a direct uptake by the liver with an absorption rate constant (*k*_a_) of 1.0 h^−1^, which is based on the fast and complete absorption of the structurally related alkenylbenzene safrole from the GI tract (Punt et al. 2008). For rat and human, the liver was the only organ able to convert myristicin to different microsomal metabolites. 2′,3′-Dihydroxymyristicin (DHM), 3′-hydroxymyristicin (3HM), 1′-hydroxymyristicin (HM), and 5-allyl-2,3-dihydroxyanisole (DHA) were formed in incubations with both rat and human liver.

Accordingly, mass balance equations for myristicin in rat liver and human were as follows:

$$\begin{aligned} &\partial {\text{AL}}_{\text{M}} /\partial t = \partial {\text{Uptake}}_{\text{M}} /\partial t + {\text{QL}} \times \left( {{\text{CA}}_{\text{M}} - {\text{CL}}_{\text{M}} /{\text{PL}}_{\text{M}} } \right) \\ & \quad - V_{{\max_{HM} }} \times {\text{CL}}_{\text{M}} /{\text{PL}}_{\text{M}} /\left( {K_{{{\text{m}}_{\text{HM}} }} + {\text{CL}}_{\text{M}} /{\text{PL}}_{\text{M}} } \right) \\ & \quad - V_{{\max_{3HM} }} \times {\text{CL}}_{\text{M}} /{\text{PL}}_{\text{M}} /\left( {K_{{{\text{m}}_{{ 3 {\text{HM}}}} }} + {\text{CL}}_{\text{M}} /{\text{PL}}_{\text{M}} } \right) \\ & \quad - V_{{\max_{DHA} }} \times {\text{CL}}_{\text{M}} /{\text{PL}}_{\text{M}} /\left( {K_{{{\text{m }}_{\text{DHA}} }} + {\text{CL}}_{\text{M}} /{\text{PL}}_{\text{M}} } \right) \\ & \quad - V_{{\max_{DHM} }} \times {\text{CL}}_{\text{M}} /{\text{PL}}_{\text{M}} /\left( {K_{{{\text{m }}_{\text{DHM}} }} + {\text{CL}}_{\text{M}} /{\text{PL}}_{\text{M}} } \right) \\ &\partial {\text{Uptake}}/\partial t = - \partial {\text{AGI}}_{\text{M}} /\partial t = K_{\text{a}} \times {\text{AGI}}_{\text{M}} , {\text{AGI}}_{\text{M}} \left( 0 \right) = {\text{oral dose}} \\& {\text{CL}}_{\text{M}} = {\text{AL}}_{\text{M}} /{\text{VL}} \\ \end{aligned}$$

where Uptake_M_ (μmol) is the amount of myristicin taken up from the GI tract, AGI_M_ (μmol) is the amount of myristicin remaining in the GI tract, and AL_M_ (μmol) is the amount of myristicin in the liver or CL_M_ is the myristicin concentration in the liver (μmol/L). CA_M_ and CV_M_ are the myristicin concentrations in the arterial and venous blood (both in μmol/L), QL is the blood flow rate to a tissue (L/h), QC is the cardiac output (L/h), VL is the volume of liver, PL_M_ is the tissue/blood partition coefficient of myristicin, and *V*_max_ and *K*_m_ are the values representing the maximum rate of formation and Michaelis–Menten constant, respectively, for the formation of 2′,3′-dihydroxymyristicin (DHM), 3′-hydroxymyristicin (3HM), 1′-hydroxymyristicin (HM), and 5-allyl-2,3-dihydroxyanisole (DHA).

The mass balance equation for the metabolic conversion of 1′-hydroxymyristicin by glucuronidation, oxidation, and sulfonation in the liver in rat and human liver is as follows:

$$\begin{aligned}& \partial {\text{AL}}_{\text{HM}} /\partial t = V_{{{ \hbox{max} }_{\text{HM}} }} \times {\text{CL}}_{\text{M}} /{\text{PL}}_{\text{M}} /\left( {K_{{{\text{m}}_{\text{HM}} }} + {\text{CL}}_{\text{M}} /{\text{PL}}_{\text{M}} } \right) \\ & \quad - V_{{{\text{max,L}}_{\text{HMG}} }} \times {\text{CL}}_{\text{HM}} /{\text{PL}}_{\text{HM}} /\left( {{\text{K}}_{{{\text{m}}_{\text{HMG}} }} + {\text{CL}}_{\text{HM}} /{\text{PL}}_{\text{HM}} } \right) \\ & \quad - V_{{{ \hbox{max} }_{\text{HMO}} }} \times {\text{CL}}_{\text{HM}} /{\text{PL}}_{\text{HM}} /\left( {K_{{{\text{m}}_{\text{HMO}} }} + {\text{CL}}_{\text{HM}} /{\text{PL}}_{\text{HM}} } \right) \\ & \quad - k_{\text{HMS}} \times {\text{CL}}_{\text{HM}} /{\text{PL}}_{\text{HM}} \\ &{\text{CL}}_{\text{M}} = {\text{AL}}_{\text{M}} /{\text{VL}} \\ \end{aligned}$$

where AL_HM_ is the amount of 1′-hydroxymyristicin in the liver (μmol), CL_HM_ is the 1′-hydroxymyristicin concentration in the liver (μmol/L), PL_HM_ is the liver/blood partition coefficient of 1′-hydroxymyristicin, and *V*_max_ and *K*_m_ are the maximum rate of formation and Michaelis–Menten constant, respectively, for the formation of the different 1′-hydroxymyristicin metabolites in the liver, including 1′-hydroxymyristicin glucuronide, 1′-oxomyristicin, and *k*_HMS_ is the first-order rate constant for sulfonation of 1′-hydroxymyristicin that was used instead of *K*_m_ and *V*_max_ as sulfonation showed no saturation. *V*_max_ and *K*_m_ values and first-order rate constants k in case of the absence of saturation (for sulfonation of 1′-hydroxymyristicin in rat and human) for the different metabolic pathways of myristicin and 1′-hydroxymyristicin were derived from results from in vitro experiments in the present study. *V*_max_ values that were derived in vitro expressed as nmol min^−1^ (mg liver microsomal or S9 protein)^−1^ were scaled to values representing the *V*_max_ per μmol h^−1^ (g liver)^−1^ using microsomal protein yields of 35 mg/g for rat and 32 mg/g for human liver and S9 protein yields of 143 mg/g for liver, as defined previously (Punt et al. 2008, 2009), based on Medinsky et al. (1994). First-order rate constant *k* expressed in ml min^−1^ (mg liver S9 protein)^−1^ was scaled to values expressed in ml h^−1^ per g liver using the same conversion factor for S9 protein yield. Tables 2 and 3 summarize the physiological parameters (i.e., tissue volumes, cardiac output, and tissue blood flows) for rat and human, respectively, which were derived from the literature (Brown et al. 1997). Partition coefficients were derived in silico based on a method described by DeJongh et al. (1997) using the log *K*_ow_. Log *K*_ow_ values for myristicin (Clog P 3.1721) and 1′-hydroxymyristicin (Clog P 1.6151) were estimated using Chemdraw professional 15 (ChemOffice^®^ Professional 15.0 by perkin elmer). Mass balance equations were coded and numerically integrated in Berkely Madonna 8.3.18 (Macey and Oster, UC Berkeley, CA) using Rosenbrock’s algorithm for stiff systems. PBK models in rat and human liver were run for 720 h, because that would be the time for total clearance of myristicin in human tissues after one dose.

**Table 2:** Parameters used in the PBK model for myristicin in male rat

Physiological parameters (Brown et al. 1997)		Tissue: blood partition coefficients	
Body weight (kg)	0.25	Myristicin	
Percentage of body weight		Liver	2.49
Liver	3.4	Fat	80.41
Fat	7.0	Rapidly perfused	2.49
Rapidly perfused	5.1	Slowly perfused	0.62
Slowly perfused	60.2		
Blood	7.4	1′-Hydroxymyristicin	
		Liver	1.12
Cardiac output (l/h)	5.4		
Percentage of cardiac output			
Liver	25		
Fat	7.0		
Rapidly perfused	51.0		
Slowly perfused	17.0

**Table 3:** Parameters used in the PBK model for myristicin in human

Physiological parameters (Brown et al. 1997)		Tissue: blood partition coefficients	
Body weight (kg)	60	Myristicin	
Percentage of body weight		Liver	6.63
Liver	2.6	Fat	105.78
Fat	21.4	Rapidly perfused	6.63
Rapidly perfused	5.0	Slowly perfused	4.18
Slowly perfused	51.7		
Blood	7.9	1′-Hydroxymyristicin	
		Liver	1.64
Cardiac output (l/h)	310		
Percentage of cardiac output			
Liver	22.7		
Fat	5.2		
Rapidly perfused	47.3		

### Sensitivity analysis

To determine which parameters have the greatest influence on model predictions, a sensitivity analysis was performed as described previously (Punt et al. 2008, 2009; van den Berg et al. 2012; Martati et al. 2011, 2012; Al-Subeihi et al. 2011, 2012). For this purpose, normalized sensitivity coefficients (SCs) were determined using the following equation:

$${\text{SC}} = \left( {C^{\prime} - C} \right)/\left( {P^{\prime} - P} \right) \times \left( {P/C} \right)$$

where *C* is the initial value of the model output, *C*′ is the modified value of the model output resulting from an increase in parameter value, *P* is the initial parameter value, and *P*′ represents the modified parameter value. An increase of 5 % in parameter values was used to analyze the effect of a change in parameter on the formation of 1′-hydroxymyristicin and 1′-sulfoxymyristicin (expressed as a percentage of the dose). Each parameter was analyzed individually, while the other parameters were kept at their initial value.

### Comparison of the PBK model-based prediction of bioactivation of myristicin to the PBK model-based predictions for bioactivation of the structurally related compound safrole

The predicted model outcomes for the formation of 1′-hydroxymyristicin and 1′-sulfoxymyristicin in the liver of rat and human were compared with the predicted dose-dependent formation of the 1′-hydroxy- and 1′-sulfoxy metabolite of the structurally related alkenylbenzene safrole. For this purpose, the previously defined PBK models for safrole described by Martati et al. (2011, 2012) for rat and human were used. For the comparison, the models describing the metabolism of myristicin and safrole were run for a period of 720 h.

## Results

### Microsomal conversion of myristicin

To identify which organs are involved in the metabolism of myristicin in both male rat and human, incubations were performed using microsomal protein preparations from liver, kidney, lung, and small intestine of both species. Chromatographic analysis of these incubations revealed that for rat and human, no detectable metabolism of myristicin occurred in small intestine, lung, or kidney microsomes, and metabolism was only observed for liver microsomes.

**Fig. 4:**
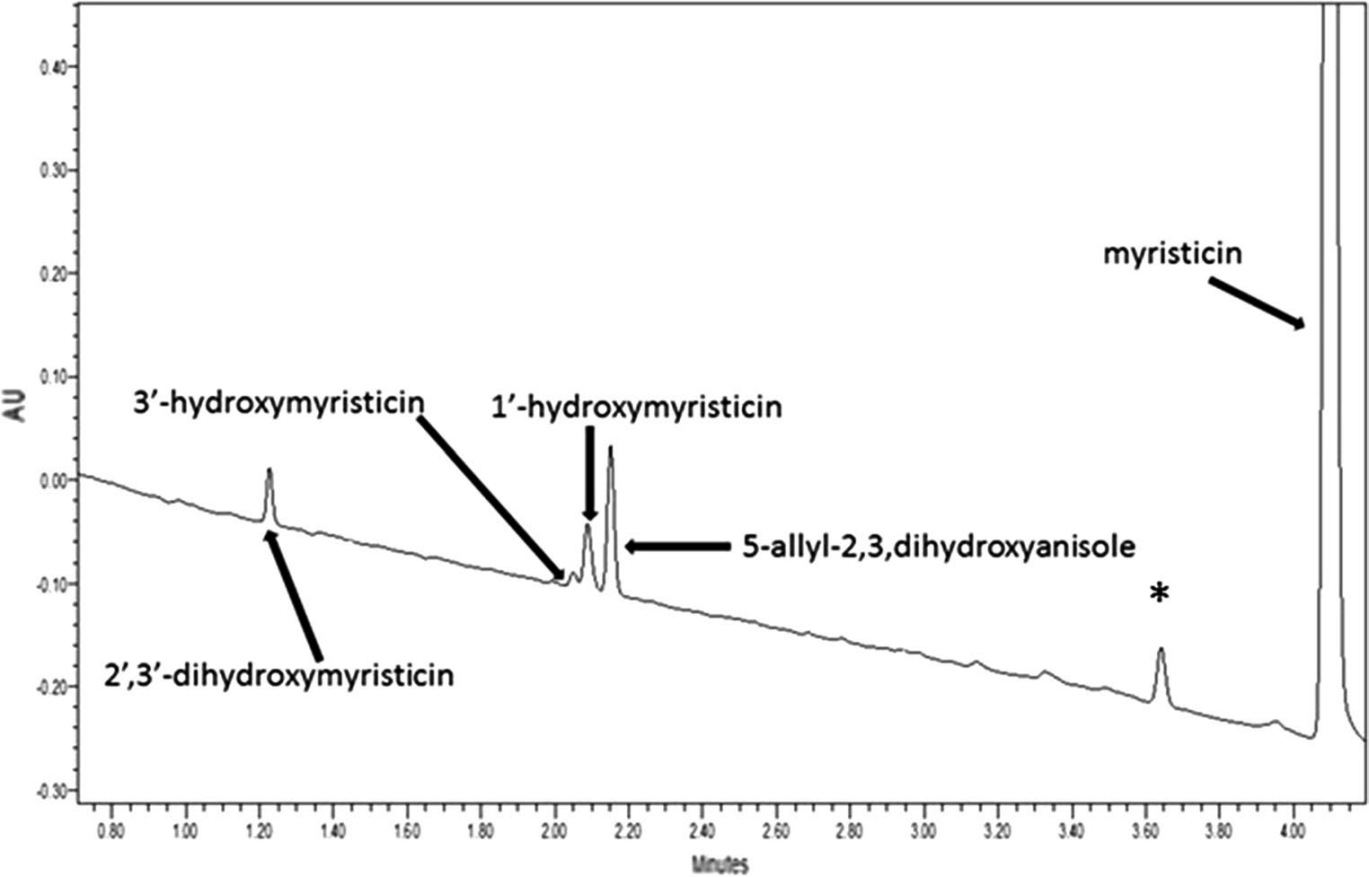
UPLC chromatogram of an incubation of myristicin (1000 µM) with male rat liver microsomes. The peak marked with an *asterisk* was also found in the blank incubation without NADPH

An example of a chromatogram of an incubation of myristicin with male rat liver microsomes and NADPH as a cofactor is shown in Fig. 4. In incubations with rat liver microsomes, 2′,3′-dihydroxymyristicin (Rt = 1.19 min), 3′-hydroxymyristicin (Rt = 2.01 min), 1′-hydroxymyristicin (Rt = 2.04 min), and 5-allyl-2,3-dihydroxyanisole (Rt = 2.11 min) were formed. In incubations with human liver microsomes, the same four metabolites were found. Identification was done based on the comparison of the UV spectra and retention times of the formed metabolites with those of the specific synthesized or commercially available reference compounds. However, tentative identification of 2′,3′-dihydroxymyrsticin was based on the fact that the epoxidation of the double bond in the allyl side chain of estragole, methyleugenol, and safrole yields the 2′,3′-epoxide, found to be hydrolyzed by epoxide hydrolase to the 2′,3′-dihydroxy metabolite (Luo et al. 1992; Luo and Guenthner 1996; Guenthner and Luo 2001). The hydrolysis product of the epoxide, 2′,3′-dihydroxy metabolite of myristicin, safrole, or elemicin, was detected in the urine of rats administered high doses (100 mg/kg bw) of each substance individually or a high dose of nutmeg (500 mg/kg bw) (Beyer et al. 2006). Data revealed that 3′-hydroxymyristicin was formed directly from myristicin rather than from isomerization of 1′-hydroxymyristicin since the formation of 3′-hydroxymyristicin was not observed in incubations of 1′-hydroxymyristicin with liver microsomes and NADPH (data not shown). The kinetic constants (i.e., *V*_max_ and *K*_m_) were derived from plots of the rates of formation of different microsomal metabolites in incubations with male rat liver microsomes, and human liver microsomes from myristicin at concentrations ranging from 25 to 1000 μM (for details, see Fig. S1 in the supplementary materials). The values obtained are shown in Table 4, together with the catalytic efficiencies, calculated as *V*_max_/*K*_m_. Analysis of incubations that were performed with male rat liver microsomal preparations revealed that the metabolite arising from O-demethylenation of myristicin, namely 5-allyl-2,3-dihydroxyanisole, was formed with the highest *V*_max_ value. Moreover, the 2′,3′-dihydroxymyristicin and 5-allyl-2,3-dihydroxyanisole were abundantly formed in incubations with male rat microsomal liver preparations with a high affinity. In incubations performed with male rat liver microsomes, 1′-hydroxymyristicin and 3′-hydroxymyristicin were the least important metabolites formed upon conversion of myristicin. In general, the catalytic efficiency for the formation of 5-allyl-2,3-dihydroxyanisole by male rat liver microsomes had the highest value, followed by the catalytic efficiency for the formation of 2′,3′-dihydroxymyristicin, 1′-hydroxymyristicin, and 3′-hydroxymyristicin, respectively. The catalytic efficiency for the formation of 5-allyl-2,3-dihydroxyanisole was found to be approximately sevenfold higher than that for the formation of 3′-hydroxymyristicin. In incubations with human liver fractions, 5-allyl-2,3-dihydroxyanisole was found to be the most abundant metabolite formed followed by 2′,3′-dihydroxymyristicin, 1′-hydroxymyristicin, and 3′-hydroxymyristicin, respectively. Analysis of the catalytic efficiencies for the formation of the different microsomal metabolites of myristicin, obtained with pooled human liver microsomes, showed that the formations of 2′,3′-dihydroxymyristicin, 1′-hydroxymyristicin, and 3′-hydroxymyristicin were the least important routes of myristicin metabolism, whereas the formation of 5-allyl-2,3-dihydroxyanisole represents the major pathway for the human liver microsomal conversion of myristicin.

**Table 4:** Kinetic constants for metabolism of myristicin and 1′-hydroxymyristicin as derived from data obtained in incubations with Sprague-Dawley male rat liver microsomes and mixed gender pooled human liver microsomes and respective cofactors

Species		Rat				Human			
Metabolite	Abbreviation	*K*_m_^a,c^	*V*_max_^a,b^	In vitro catalytic efficiency ^d^	*k* ^f^ ± SD	*K*_m_^a,c^	*V*_max_^a,b^	In vitro catalytic efficiency^d^	*k* ^f^ ± SD
		Conversion of myristicin							
2′,3′-Dihydroxymyristicin	DHM	16 ± 7	0.44 ± 0.08	27.5		33 ± 12	0.08 ± 0.01	2.424	
3′-Hydroxymyristicin	3HM	83 ± 31	0.47 ± 0.09	5.663		1629 ± 1545	0.29 ± 0.14	0.178	
1′-Hydroxymyristicin	HM	102 ± 32	0.86 ± 0.18	8.431		234 ± 132	0.17 ± 0.03	0.726	
5-Allyl-2,3-dihydroxyanisole	DHA	33 ± 11	1.3 ± 0.23	39.394		118 ± 36	0.93 ± 0.13	7.881	
		Conversion of 1′-hydroxymyristicin							
1′-Hydroxymyristicin glucuronide^e^	HMG	836 ± 336	8.6 ± 2.0	10.287		3140 ± 2434	0.08 ± 0.03	0.025	
1′-Oxomyristicin	HMO	5922 ± 3477	43.64 ± 20.9	7.369		3244 ± 2646	32 ± 14	9.864	
1′-Sulfoxymyristicin^e^	HSM				17.6 × 10^−6^				16.3 × 10^−6^

^a^Mean values of three independent measurements ± SD

^b^nmol min^−1^ (mg microsomal or S9 protein)^−1^

^c^μM

^d^μl min^−1^ (mg microsomal or S9 protein)^−1^ (*V*_max_/*K*_m_ × 1000 μL/ml)

^E^Experiments performed with S9 tissue fractions. First-order rate constant for product formation(ml/min/mg protein)

### Glucuronidation of 1′-hydroxymyristicin

**Fig. 5:**
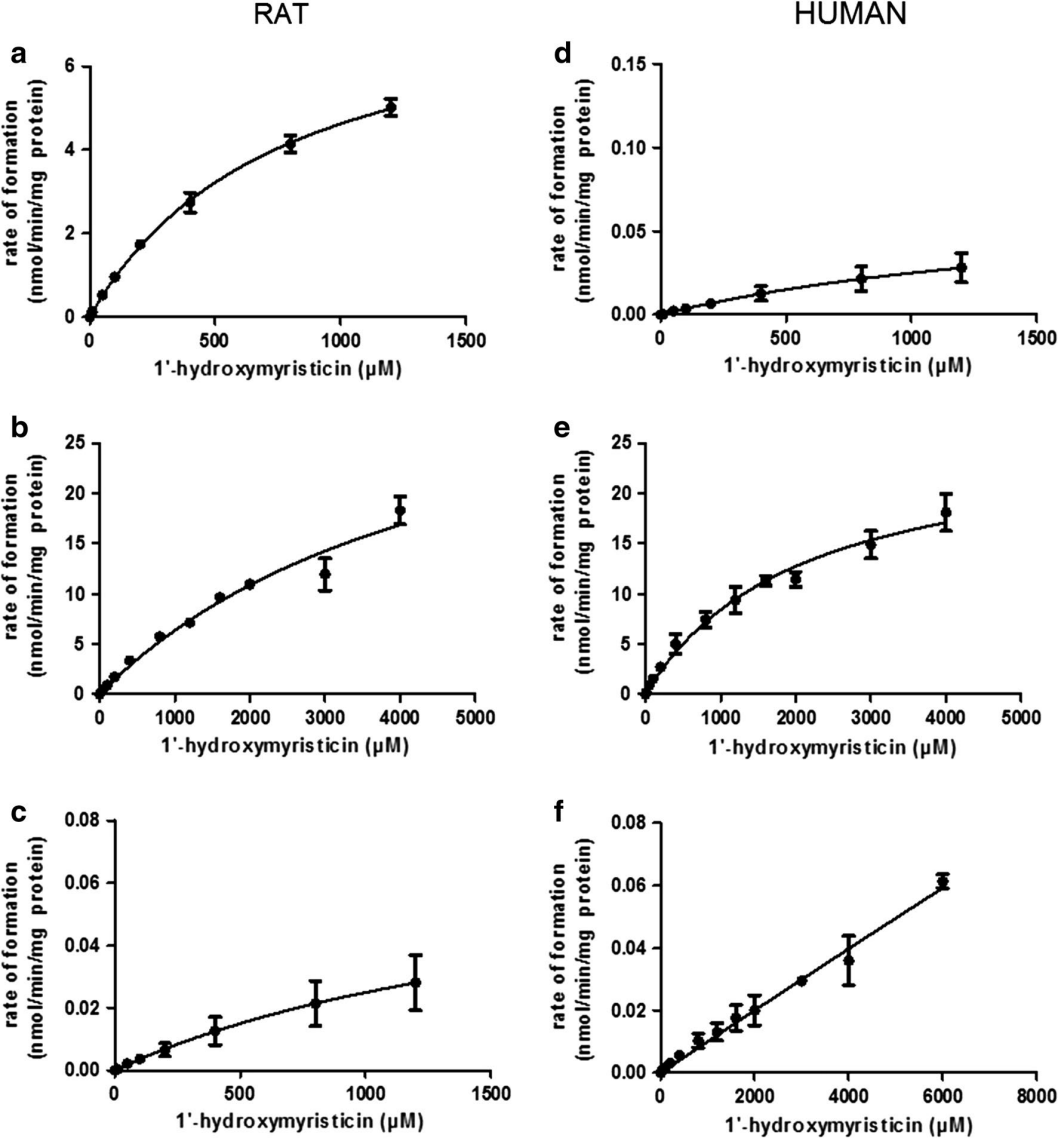
Concentration-dependent rate of **a** glucuronidation of 1ʹ-hydroxymyristicin in incubations with male rat liver S9 or **d** pooled human mixed gender liver S9, **b** oxidation of 1ʹ-hydroxymyristicin in incubations with pooled male rat liver microsomes or **e** pooled human mixed gender liver S9, and **c** sulfonation of 1ʹ-hydroxymyristicin in pooled male rat liver S9 or **f** pooled human mixed gender liver S9. *Data points* represent mean values ±SD of three or four individual experiments

Chromatographic analysis of incubations with male rat and mixed gender pooled human liver S9, UDPGA as cofactor and 1′-hydroxymyristicin as substrate, revealed a peak at 1.36 min (chromatogram not shown). Moreover, chromatographic analysis of incubations performed in the absence of the cofactor UDPGA did not show a peak at a retention time of 1.36 min. Together, these data indicate that the compound eluting at 1.36 min can be assumed to be 1′-hydroxymyristicin glucuronide. The rate of the metabolic conversion of 1′-hydroxymyristicin to 1′-hydroxymyristicin glucuronide in incubations with both male rat and human liver fractions with increasing concentrations of 1′-hydroxymyristicin is presented in Fig. 5a, d, respectively. The kinetic constants derived from these plots are presented in Table 4.

### Oxidation of 1′-hydroxymyristicin

The rate of oxidation of 1′-hydroxymyristicin with increasing concentrations of 1′-hydroxymyristicin in incubations with male rat and pooled human liver microsomes is shown in Fig. 5b, e, respectively, and the kinetic constants derived from these data are presented in Table 4.

### Sulfonation of 1′-hydroxymyristicin

In the present study, GSH was used to trap the reactive 1′-sulfoxymyristicin formed upon sulfonation of the proximate carcinogenic metabolite of myristicin, 1′-hydroxymyristicin. The scavenging is based on a chemical reaction but can also be catalyzed by the GST present in the S9 incubations in which the sulfonation of 1′-hydroxymyristicin was measured. Chromatographic analysis of incubations with male rat or mixed gender pooled human liver S9, increasing concentration of 1′-hydroxymyristicin, PAPS, and GSH revealed a peak at 1.05 min, which was identified as the GSH adduct of the carbocation of 1′-sulfoxymyristicin. Identification was achieved based on the chromatographic analysis of incubations performed in the absence of GSH and the presence of PAPS and liver S9 proteins since in these incubations, no peak was found at 1.05 min. The identity was verified by LC–MS analysis, which revealed a deprotonated molecule at *m/z* 496 that corresponds to the expected [M–H]^−^ mass of a GSH adduct of 1′-sulfoxymyristicin. The rate of formation of 1′-sulfoxymyristicin in incubations with male rat and mixed gender pooled human liver S9 is presented in Fig. 5c, f, respectively. The kinetic constants for the conversion of 1′-hydroxymyristicin to 1′-sulfoxymyristicin in rat and human liver are presented in Table 4.

### Comparison of the kinetic constants for conversion of myristicin and 1′-hydroxymyristicin by male rat and mixed gender pooled human tissue fractions

To allow a comparison between the kinetic constants for metabolism of myristicin and 1′-hydroxymyristicin by male rat and mixed gender pooled human tissue fractions, *V*_max_ values that were derived in vitro expressed in nmol min^−1^ (mg microsomal or S9 protein)^−1^ were scaled to values representing the *V*_max_ per μmol h^−1^ (g tissue)^−1^ using microsomal and S9 protein yields as described in the literature (Medinsky et al. 1994) and previously used (Martati et al. 2011, 2012; van den Berg et al. 2012; Punt et al. 2009; Al-Subeihi et al. 2011, 2012). Making the use of the in vivo *V*_max_ values derived accordingly, a scaled catalytic efficiency (scaled $V_{ \hbox{max} } {\text{in vivo}}/K_{\text{m}}$) for the formation of myristicin metabolites could be calculated (Table 5). For sulfonation, the rate constants for product formation (ml/min/mg protein) were scaled based on S9 protein yield of 143 mg/g liver. These values show that the catalytic efficiency for the formation of the proximate carcinogenic metabolite of myristicin, 1′ hydroxymyristicin, was found to be 13-fold higher in male rat liver as compared to human liver. This difference in catalytic efficiency for the formation of 1′-hydroxymyristicin is predominantly caused by the high affinity (expressed as *K*_m_) and high activity (expressed as *V*_max_) for its formation from myristicin in male rat liver, since the *K*_m_ value in rat liver incubations was twofold lower than that in human liver incubations and *V*_max_ in rat liver incubations was fivefold higher than that in human liver incubations. The detoxification of 1′-hydroxymyristicin by formation of 1′-hydroxymyristicin glucuronide was found to be the main metabolic reaction with 1′-hydroxymyristicin in rat. Glucuronidation of 1′-hydroxymyristicin occurs in male rat with higher affinity than in human (i.e., *K*_m_ 836 μM), whereas in human the *K*_m_ was 3140 μM, and analysis of the data revealed a high *V*_max_ value for 1′-hydroxymyristicin glucuronidation resulting in a catalytic efficiency that was 400-fold higher in male rats as compared to human. Oxidation of 1′-hydroxymyristicin was found to be 1.2 times more efficient in human liver as compared to male rat liver resulting from a 1.8-fold lower affinity in rat. Sulfonation was found to be the least efficient metabolic pathway for 1′-hydroxymyristicin in both rats and human. For rat, the in vivo scaled *k* was 0.15 ml h^−1^ (g tissue)^−1^, and for human, it was 0.14 ml h^−1^ (g tissue)^−1^, indicating that sulfonation of 1′-hydroxymyristicin is equally efficient in male rat liver and human liver. Altogether, it can be concluded that glucuronidation of 1′-hydroxymyristicin, representing a detoxification pathway, is the most important pathway in rat, and the oxidation is the most important pathway for conversion of 1′-hydroxymyristicin in human. Moreover, on the basis of the kinetic data obtained, bioactivation of 1′-hydroxymyristicin following sulfonation was found to represent only a minor pathway in both rat and human.

**Table 5:** Scaled kinetic constants for metabolic conversion of myristicin and 1′-hydroxymyristicin by male rat and human tissue fractions

Species		Rat^a^				Human^a^			
Metabolite	Abbreviation	Scaled *V*_max_, in vivo (μmol/h/g tissue)^b^	*K*_m_ (uM)	In vivo catalytic efficiency (ml/h/g tissue)^c^	*k* Scaled (ml/hr/g tissue)^d^	Scaled *V*_max_, in vivo (μmol/h/g tissue)^b^	*K*_m_ (uM)	In vivo catalytic efficiency (ml/h/g tissue)^c^	*k* Scaled (ml/hr/g tissue)^d^
		Conversion of myristicin							
2′,3′-Dihydroxymyristicin	DHM	0.924	16 ± 7	0.0578		0.154	33 ± 12	0.0047	
3′-Hydroxymyristicin	3HM	0.987	83 ± 31	0.0119		0.557	1629 ± 1545	0.0003	
1′-Hydroxymyristicin	HM	1.806	102 ± 32	0.0177		0.326	234 ± 132	0.0014	
5-Allyl-2,3-dihydroxyanisole	DHA	2.73	33 ± 11	0.0827		1.786	118 ± 36	0.0151	
		Conversion of 1′-hydroxymyristicin							
1′-Hydroxymyristicin glucuronide	HMG	73.788	836 ± 336	0.0883		0.6864	3140 ± 2434	0.0002	
1′-Oxomyristicin	HMO	91.644	5922 ± 3477	0.0155		61.44	3244 ± 2646	0.0189	
1′-Sulfoxymyristicin	HMS				0.15				0.14

^a^Mean values of three independent measurements

^b^Scaled *V*_max_ values were converted from in vitro *V*_max_ values based on microsomal protein yields of 35 and 32 mg/g tissue liver for rat and human, respectively, and a S9 protein yield of 143 mg/g liver

^c^Catalytic efficiency(scaled *V*_max_ (app)/*K*_m_)

^d^ *k* Scaled based on S9 protein yield of 143 mg/g liver

### Evaluation of PBK model performance

The performance of the newly developed PBK models for myristicin could not be evaluated against in vivo data because quantitative data on the formation or excretion of the different metabolites in rat or humans exposed to myristicin are not available. However, Beyer et al. (2006) reported that in rats and humans, myristicin and safrole were O-demethylenated. In the urine of the human nutmeg abuser, who had taken 5 nutmegs, the acetylated metabolites of 5-allyl-2,3-dihydroxyanisole and 2′,3′-dihydroxymyristicin could be identified. In the same study, O-demethylenation of myristicin to generate 5-allyl-2,3-dihydroxyanisole seems to result in the main metabolite of myristicin detected in rat urine samples collected over a 24-h period after administration of a single oral dose of 100 mg/kg bw myristicin, with a percentage of 67 % of the total dose (Beyer et al. 2006). In line with these results, the developed PBK model predicted 5-allyl-2,3-dihydroxyanisole to be the major metabolite formed at a dose of 100 mg/kg bw myristicin in rat after 24 h, with a percentage of 73 % of the total dose. This predicted value of 73 % of the dose matches well with the 67 % observed in the in vivo rat study.

Important to note is that the PBK models for myristicin were based on the proposed biotransformation in Fig. 2 and comparable with the PBK models for estragole, methyleugenol, and safrole, for which more data allowing the evaluation of the models were available. The performance of the rat PBK models developed for estragole, methyleugenol, and safrole was reported before (Punt et al. 2008; Martati et al. 2011; Al-Subeihi et al. 2011). Evaluation was done by comparing the predicted levels of a variety of metabolites in plasma or excreted in the urine of rats. Data revealed that the predicted PBK model values and the levels of these metabolites derived from in vivo studies adequately matched (Punt et al. 2008; Martati et al. 2011; Al-Subeihi et al. 2011). Furthermore, also for the developed human PBK models for estragole (Punt et al. 2009), methyleugenol (Al-Subeihi et al. 2012), and safrole (Martati et al. 2012), a comparison could be made between the model predictions and the reported in vivo data for blood concentrations or the urinary excretion of some of the metabolites, thereby further supporting the validity of the models. Considering these data, it was concluded that the developed PBK models for myristicin will also adequately describe the in vivo levels of metabolites formed in rat and human after conversion of myristicin and 1′-hydroxymyristicin at different oral dose levels of myristicin.

### PBK model predictions

**Fig. 6:**
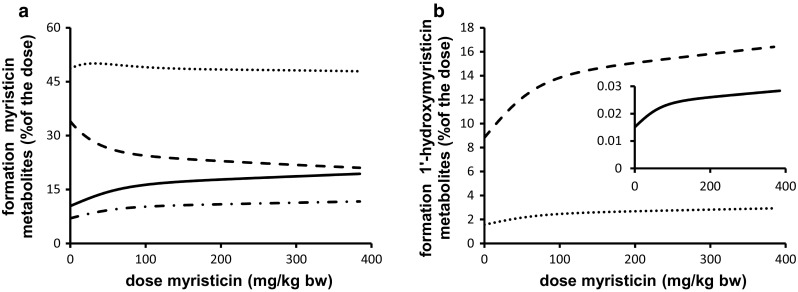
PBK-predicted dose-dependent changes in overall formation of **a** microsomal metabolites of myristicin in rat liver and **b** metabolites of 1′-hydroxymyristicin in rat liver. The *lines* correspond to (**a**) 5-allyl-2,3-dihydroxyanisole (*dotted line*), 3′-hydroxymyristicin (*dashed and* *dotted line*), 1′-hydroxymyristicin (*straight line*), 2′,3′-dihydroxymyristicin (*dashed line*), (B) 1′-hydroxymyristicin glucuronide (*dashed lines*), 1′-sulfoxymyristicin (*straight line*), and 1′-oxomyristicin (*dotted line*)

**Fig. 7:**
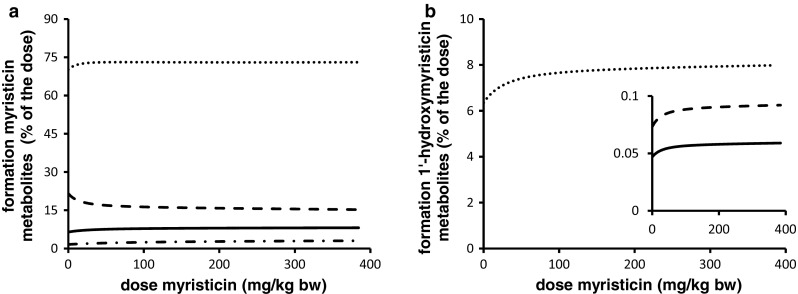
PBK model-based prediction of dose-dependent changes in overall formation of (**a**) microsomal metabolites of myristicin in human liver and (**b**) metabolites of 1′-hydroxymyristicin in human liver. The lines correspond to (**a**) 5-allyl-2,3-dihydroxyanisole (*dotted line*), 3′-hydroxymyristicin (*dashed and* *dotted line*), 1′-hydroxymyristicin (*straight line*), 2′,3′-dihydroxymyristicin (*dashed line*), (**b**) 1′-hydroxymyristicin glucuronide (*dashed line*), 1′-sulfoxymyristicin (*straight line*), and 1′-oxomyristicin (*dotted line*)

**Fig. 8:**
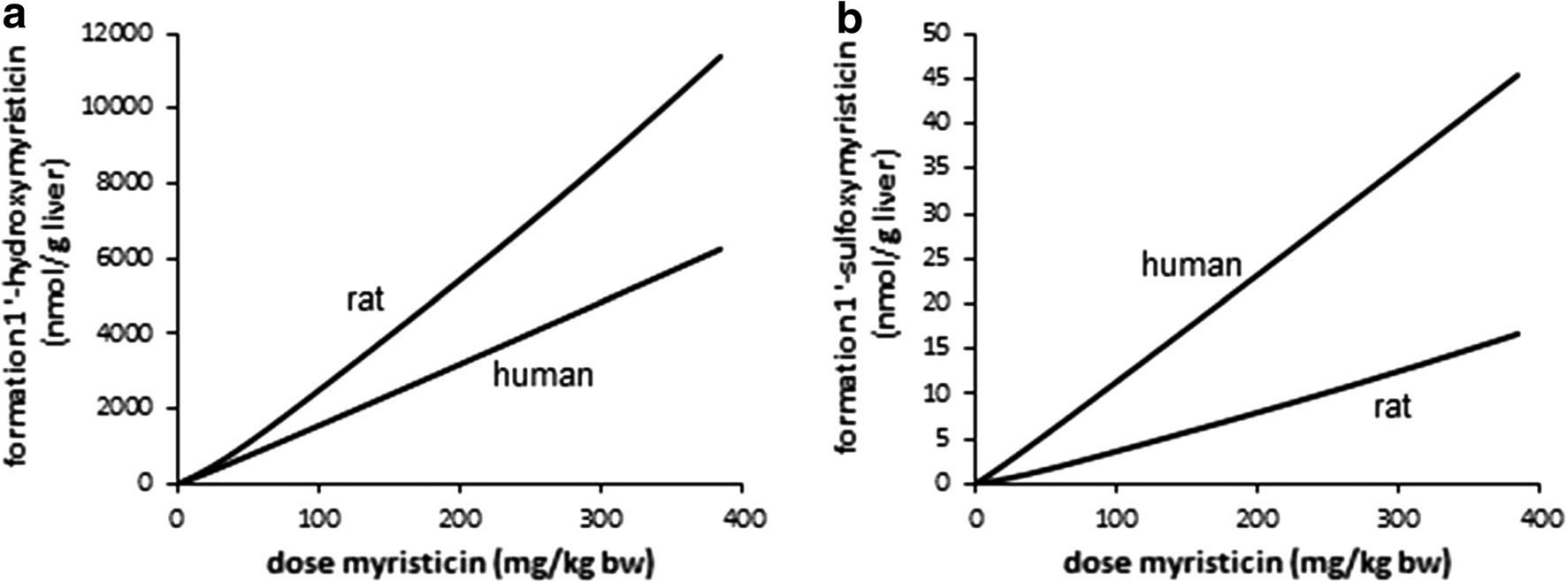
PBK model-based prediction of dose-dependent formation (mg/kg bw) of **a** 1′-hydroxymyristicin and **b** 1′-sulfoxymyristicin in rat and human liver

PBK modeling was performed at dose levels of 0.05 and 300 mg/kg bw to allow comparison with the PBK model outcomes previously reported for safrole (Martati et al. 2011, 2012). Following an exposure to 0.05 mg/kg bw myristicin, both myristicin and its proximate carcinogenic metabolite 1′-hydroxymyristicin were predicted and observed to be almost completely metabolized within a 720-h period in rat and human. At a higher oral dose level of 300 mg/kg bw myristicin, both myristicin and 1′-hydroxymyristicin were also predicted to be fully metabolized within 720 h. Therefore, for all further modelings, values were calculated at 720 h after dosing. Figure 6a shows the PBK model-based predictions for the dose-dependent formation of the different microsomal metabolites of myristicin in rat. The percentage of the dose converted to 1′-hydroxymyristicin is predicted to increase in a dose-dependent manner. Concurrent with the increased percentage of the dose that undergoes 1′-hydroxylation of the alkene side chain, a 1.6-fold dose-dependent decrease in the percentage of the dose that underwent epoxidation to give 2′,3′-dihydroxymyristicin was observed comparing the levels at 0.05 and 300 mg/kg bw. At the same time, a <1.6-fold increase in the formation of 3′-hydroxymyristicin was observed. The formation of 5-allyl-2,3-dihydroxyanisole did not change with increasing dose levels and was equal to 48 % of the administered dose. Figure 6b shows the dose-dependent increase in the formation of the metabolites of 1′-hydroxymyristicin in rat. This reveals a 1.8-fold increase in the percentage of the dose ultimately converted into 1′-hydroxymyristicin glucuronide, 1′-oxomyristicin, and 1′-sulfoxymyristicin, going from a dose of 0.05 to 300 mg/kg bw. This dose-dependent increase in the formation of the different 1′-hydroxymyristicin metabolites can be explained by the 1.8-fold increase in the formation of 1′-hydroxymyristicin with increasing dose levels. Figure 7a reveals that in human, the percentage of the dose converted to 1′-hydroxymyristicin equaled 6.5 % at a dose of 0.05 mg/kg bw and increased to 8.0 % at a dose of 300 mg/kg bw. Accompanying the increase in the formation of 1′-hydroxymyristicin, there were a 1.4-fold decrease in the formation of 2′,3′-dihydroxymyristicin and a steady formation of 5-allyl-2,3-dihydroxyanisole. Formation of 3′-hydroxymyristicin was predicted to increase 1.8-fold with increasing dose from of 0.05 to 300 mg/kg bw. Figure 7b shows a 1.25-fold dose-dependent increase in the percentage formation of 1′-hydroxymyristicin glucuronide, 1′-sulfoxymyristicin, and 1′-oxomyristicin, in human liver with increasing dose from of 0.05 to 300 mg/kg bw. Comparison of the relative extent of bioactivation of myristicin by rat and human liver revealed that formation of 1′-hydroxymyristicin (expressed as nmol/g liver) is comparable in rat and human liver at the low dose of 0.05 mg/kg bw and 1.8-fold higher in rat liver than in human liver at a dose of 300 mg/kg bw (Fig. 8a). Formation of 1′-sulfoxymyristicin (expressed as nmol/g liver) is fourfold higher in human liver than in rat liver at a low dose of 0.05 mg/kg bw and 2.8-fold lower in rat liver than in human liver at a dose of 300 mg/kg bw (Fig. 8b).

### Sensitivity analysis

**Fig. 9:**
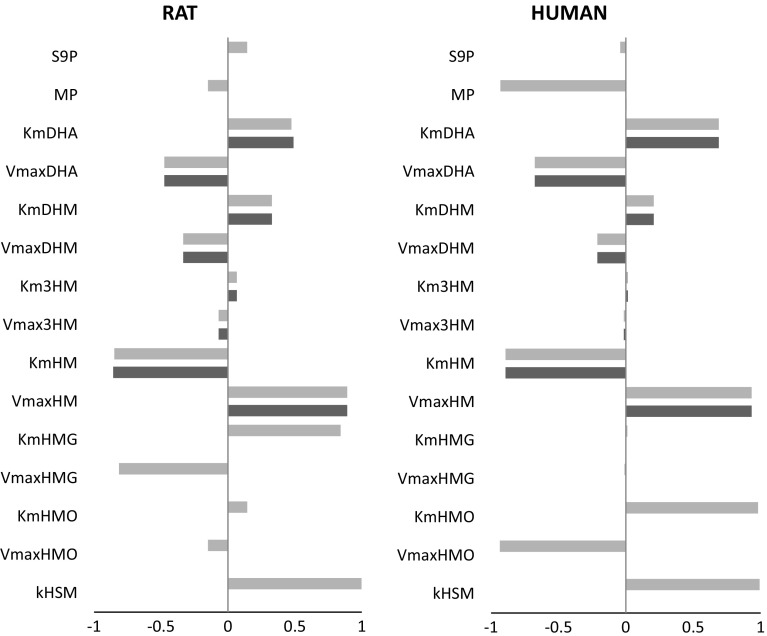
Normalized sensitivity coefficients for the formation of 1′-hydroxymyriticin (*black*) and 1′sulfoxymyristicin (*gray*) in the liver of rat or human

A sensitivity analysis was performed to define model parameters that are capable of influencing the formation of 1′-hydroxymyristicin and 1′-sulfoxymyristicin in rat and human liver. For this purpose, normalized sensitivity coefficients (SCs) were calculated for all parameters at a dose of 0.05 mg/kg bw myristicin. This sensitivity analysis reveals to what extent small variation in the respective parameters influences the results. The sensitivity analysis also reveals to which parameters the predicted outcomes are most sensitive. The results of this analysis are presented in Fig. 9. Figure 9 especially presents the parameters affecting the formation of 1′-hydroxymyristicin (black) and 1′-sulfoxymyristicin (gray) that have a normalized SC >|0.1|. In rat liver, the formation of the ultimate carcinogenic metabolite 1′-sulfoxymyristicin is primarily influenced by the kinetic constants of 1′-hydroxymyristicin formation from myristicin ($V_{{\max_{\text{HM}} }} ,\;K_{{{\text{m}}_{\text{HM}} }}$). The kinetic constants for the formation of 5-allyl-2,3-dihydroxyanisole and 2′,3′-dihydroxymyristicin ($V_{{\max_{\text{DHA}} }} ,\;K_{{{\text{m}}_{\text{DHA}} }} ,\;V_{{\max_{\text{DHM}} }} ,\;K_{{{\text{m}}_{\text{DHM}} }}$) were also found to highly influence the formation of 1′-hydroxymyristicin in rat and human liver. In rat liver, the formation of the ultimate carcinogenic metabolite 1′-sulfoxymyristicin is primarily influenced by the kinetic constants of 1′-hydroxymyristicin formation from myristicin ($V_{{\max_{\text{HM}} }} ,\;K_{{{\text{m}}_{\text{HM}} }}$), and the kinetic constants for the formation of 1′-hydroxymyristicin glucuronide ($V_{{\max_{\text{HMG}} }} ,\;K_{{{\text{m}}_{\text{HMG}} }}$), *k*, the first-order rate constant for the sulfonation of 1′-hydroxymyristicin (*k*_HMS_), and the kinetic constants for the formation of 5-allyl-2,3-dihydroxyanisole and 2′,3′-dihydroxymyristicin ($V_{{\max_{\text{DHA}} }} ,\;K_{{{\text{m}}_{\text{DHA}} }} ,\;V_{{\max_{\text{DHM}} }} ,\;K_{{{\text{m}}_{\text{DHM}} }}$) were also found to highly influence the formation of 1′-sulfoxymyristicin in rat liver. In human liver, the formation of the ultimate carcinogenic metabolite 1′-sulfoxymyristicin is primarily influenced by the kinetic constants of 1′-hydroxymyristicin formation from myristicin ($V_{{\max_{\text{HM}} }} ,\;K_{{{\text{m}}_{\text{HM}} }}$), the kinetic constants for the formation of 1′-oxomyristicin ($V_{{\max_{\text{HMO}} }} ,\;K_{{{\text{m}}_{\text{HMO}} }}$), *k*, the first-order rate constant for the sulfonation of 1′-hydroxymyristicin (*k*_HMS_) and liver microsomal protein yield (MP), and the kinetic constants for the formation of 5-allyl-2,3-dihydroxyanisole and 2′,3′-dihydroxymyristicin ($V_{{\max_{\text{DHA}} }} ,\;K_{{{\text{m}}_{\text{DHA}} }}$) were also found to highly influence the formation of 1′-sulfoxymyristicin in human liver. The kinetic constants for the formation of 1′-hydroxymyristicin glucuronide were found to highly influence the formation of 1′-sulfoxymyristicin in rat liver, and the kinetic constants for formation of 1′-oxomyristicin were predicted to affect the formation of 1′-sulfoxymyristicin in human liver to a high extent. These results reflect the fact that glucuronidation of 1′-hydroxymyristicin in rat and oxidation of 1′-hydroxymyristicin in human are considered as the most important competitive metabolic pathways to sulfonation.

### Comparison of the PBK model-based prediction of bioactivation of myristicin by rat and human to that of its structurally related compound safrole

**Fig. 10:**
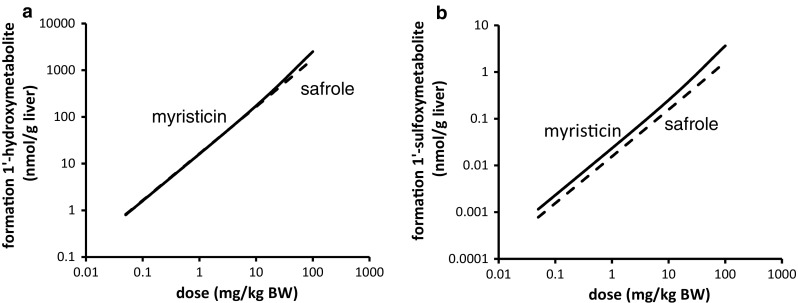
PBK model-based prediction of dose-dependent formation (nmol/g liver) of **a** 1′-hydroxy metabolites and **b** 1′-sulfoxy metabolite of myristicin (*straight line*) and safrole (*dashed line*) in male rat liver

**Fig. 11:**
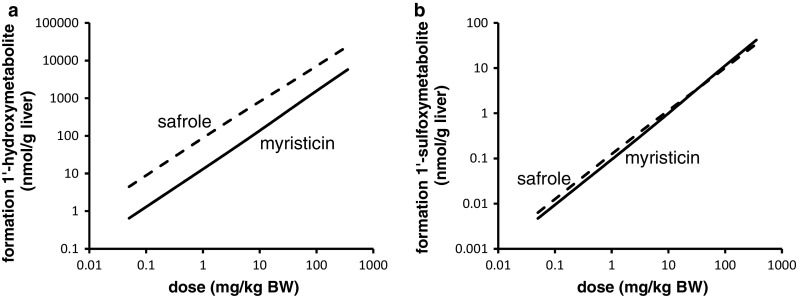
PBK model-based prediction of dose-dependent formation (nmol/g liver) of **a** 1′-hydroxy metabolites and **b** 1′-sulfoxy metabolites of myristicin (*straight line*) and safrole (*dashed line*) in human liver

In a next step, the mode of action-based PBK models for myristicin metabolism in rat and human were used to facilitate a read-across from data on safrole (Martati et al. 2011, 2012). On the basis of the PBK models, a comparison was made for the dose-dependent formation of the proximate carcinogenic 1′-hydroxy metabolite and of the ultimate carcinogenic 1′-sulfoxy metabolite of myristicin and safrole in the liver of rats. Figure 10 shows the dose-dependent formation of these metabolites in rat liver as predicted by the respective PBK models. The PBK model-based predicted formation of the proximate carcinogenic 1′-hydroxy metabolites shows that the formation of the 1′-hydroxy metabolites of myristicin and safrole was predicted to be the same at low dose 0.05 mg/kg bw and 1.4-fold higher for myristicin than that of safrole at dose level 100 mg/kg bw (Fig. 10a). The predicted model outcomes for the formation of the ultimate carcinogenic 1′-sulfoxy metabolites of safrole and myristicin are shown in Fig. 10b. The PBK models for rat predict the formation of 1′-sulfoxymyristicin to be 1.5-fold higher for myristicin than safrole at low dose of 0.05 mg/kg bw and 2.2-fold higher for myristicin than for safrole at dose level of 100 mg/kg bw. Figure 11 shows the predicted dose-dependent formation of 1′-hydroxy metabolites and 1′-sulfoxy metabolites of safrole and myristicin in human liver. In human liver, the predicted formation of 1′-hydroxymyristicin is sevenfold lower than the formation of 1′-hydroxysafrole at a dose level of 0.05 mg/kg bw and 4.5-fold lower for myristicin than for safrole at high dose of 100 mg/kg bw (Fig. 11a). The data also reveal that in human liver, the formation of the DNA-reactive 1′-sulfoxy metabolite is comparable, 1.35-fold higher for safrole at low-dose level 0.05 mg/kg bw, and 1.1-fold higher for myristicin at high-dose level 100 mg/kg bw (Fig. 11b).

### Implications for risk assessment

The margin-of-exposure (MOE) concept was applied to assess the possible risks for human health resulting from the daily exposure to myristicin (JECFA 2005; EFSA 2005; Barlow et al. 2006; O’Brien et al. 2006). The MOE is a dimensionless ratio based on a reference point representing a dose causing a low but measurable cancer incidence in experimental animals (e.g., a BMDL_10_), which is divided by the estimated daily intake in humans (EFSA 2005). When the MOE is lower than 10,000, the compound of interest is considered to be a priority for risk management actions and a concern for human health (EFSA 2005). To date, tumor data for myristicin, from which a BMDL_10_ can be derived, are absent in the available literature, hampering the application of the MOE approach in the risk assessment of myristicin. Nevertheless, the results of the PBK model predictions presented above indicate that at dose levels in the range of the BMDL_10_ values, the formation of 1′-sulfoxy metabolites of myristicin in human liver is comparable (1.1-fold higher) to that of the structurally related safrole. On the basis of these considerations, and the limited difference in 1′-sulfoxy metabolite formation for myristicin and safrole in rat and human liver, it was concluded that the BMDL_10_ values of safrole could be used to perform an initial MOE-based risk assessment for myristicin. Using the BMDL_10_ for safrole of 1.9–5.1 mg/kg bw per day (van den Berg et al. 2011) and an estimated daily intake of myristicin of 0.0019 mg/kg bw per day (WHO 2009) from use of herbs and spices, the MOE for myristicin would amount to 1000–2684. The estimated daily intake of safrole from spice and spice oil in the USA amounts to 0.001 mg/kg bw per day (WHO 2009). These data result in an MOE for safrole of 1900–5100, indicating a priority for risk management that is somewhat lower than for myristicin.

## Discussion

In the presented paper, the recently developed mode of action-based PBK models for detoxification and bioactivation of the alkenylbenzene safrole (Martati et al. 2011, 2012) in male rat and human were extended to the structurally related alkenylbenzene myristicin. The newly developed PBK models combine biochemical and physicochemical information on myristicin and on the physiology of the organism of interest (i.e., rat and human), enabling the quantification of detoxification and bioactivation in rat and human at realistic low exposure levels. The development of these models facilitates a risk assessment based on read-across from data on safrole, for which in vivo chronic toxicity studies are available, to myristicin, a compound for which toxicity data are limited.

The PBK models for myristicin defined in the present paper were able to predict the overall formation of the reactive 1′-sulfoxymyristicin metabolite in the liver of rat and human, thus enabling comparison to the overall formation of the 1′-sulfoxy metabolites of the corresponding alkenylbenzene safrole. Comparison of the rat and human PBK model predictions indicated an only limited species-dependent difference in the overall metabolic activation of myristicin. The difference observed was within the default factor of 4, which is generally used to describe kinetic differences between species (IPCS 2010). The newly developed PBK model for myristicin was also used to compare the levels of metabolic activation of myristicin to those predicted previously for safrole in male rat and human liver (Martati et al. 2011, 2012). Results reveal that the formation of the proximate and ultimate carcinogenic metabolites of myristicin and safrole in rat liver appears to be comparable varying about 1.4-fold and 2.2-fold, respectively, with rat liver bioactivation of myristicin predicted to be somewhat higher than that of safrole for both metabolites. In humans, the formation of especially the 1′-sulfoxy metabolites of the two alkenylbenzenes is predicted to be comparable (1.1-fold difference). The PBK model outcomes obtained for the formation of reactive 1′-sulfoxy metabolites of myristicin and safrole can be compared to the relative bioactivation of these two alkenylbenzenes observed in other studies. In an in vitro study with cultured human cells, the ability of myristicin to form DNA adducts upon exposure of the cells to myristicin appeared to be almost the same as upon exposure to safrole (Zhou et al. 2007b) which is in line with the relative differences predicted by the PBK models. Data derived from another study in which mice were exposed to the alkenylbenzenes via intraperitoneal (i.p) injection (Table 1) suggest that the DNA adduct formation of safrole and myristicin is also not much different with DNA adduct formation in the liver of safrole being 2.7 times higher than that of myristicin (Randerath et al. 1984). In a parallel study, Phillips et al. (Table 1) reported that in neonatal mice, both safrole and myristicin were able to form DNA adducts in liver, and the DNA binding levels of safrole and myristicin were 17.5 and 7.8 pmol/mg DNA, respectively (Phillips et al. 1984). It is important to note that in these mice studies, DNA adduct levels were quantified by ^32^P post-labeling which is known to be less accurate than, for example, LC–MS in the quantification of DNA adduct levels (Randerath et al. 1984). On the basis of the results now available, it can be concluded that using data on safrole for a read-across to myristicin is a reasonable approach for an initial risk assessment on myristicin. Such a risk assessment for myristicin can be based on the MOE approach. Because data on tumor formation are currently not available for myristicin, risk assessment for myristicin was performed using the BMDL_10_ values for tumor formation by safrole, given the comparable bioactivation in human liver predicted by the newly developed PBK models, where the difference in the bioactivation of myristicin and safrole was predicted to be only 1.1-fold.

Using the BMDL_10_ for safrole of 1.9–5.1 mg/kg bw per day (van den Berg et al. 2011) and an estimated daily intake of myristicin of 0.0019 mg/kg bw per day (WHO 2009) from use of herbs and spices, the MOE for myristicin would amount to 1000–2684. For comparison, the MOE values of safrole can be given, obtained at the estimated daily intake of safrole from spice and spice oil in the USA that amounts to 0.001 mg/kg bw per day (WHO 2009) and a BMDL_10_ of safrole of 1.9–5.1 mg/kg bw per day (van den Berg et al. 2011). These data result in an MOE for safrole of 1900–5100, indicating a priority for risk management that is lower than for myristicin.

Altogether, the results obtained indicate that PBK modeling provides an important insight into the limited species differences between male rat and human in the metabolic activation of myristicin, and that in human liver, formation of the ultimate carcinogenic 1′-sulfoxy metabolites is almost the same for myristicin and safrole supporting a possibility for the risk assessment for myristicin based on the MOE approach using the BMDL_10_ for tumor formation of safrole as a reasonable but careful approximation. The present study provides an example of how PBK modeling can facilitate a read-across in risk assessment from compounds for which in vivo toxicity studies are available to a compound for which only limited toxicity data have been described, thus contributing to the development of alternatives for animal testing.

## Electronic supplementary material

Below is the link to the electronic supplementary material.

Supplementary material 1 (DOCX 67 kb)
