# Supplementary material for: Physiologically based kinetic modeling of the bioactivation of myristicin
Source: Arch Toxicol. 2016 Jun 22;91(2):713–34. doi: 10.1007/s00204-016-1752-5 (PMC5306082; doi:10.1007/s00204-016-1752-5)
Supplement: Supplementary file 1 — Supplementary material 1 (DOCX 67 kb) [file 204_2016_1752_MOESM1_ESM.docx]

**Archives of Toxicology**

Ref.:  Ms. No. ATOX-D-16-00114

**Physiologically based kinetic modeling of the bioactivation of myristicin.**

Amer J. Al-Malahmeh^1,2^, Abdelmajeed Al-Ajlouni^1,2^, Sebastiaan Wesseling^1^, Ans E.M. F. Soffers^1^, Ala’Al-Subeihi^4^, Reiko Kiwamoto^1^, Jacques Vervoort^3^, Ivonne M.C.M. Rietjens^1^

^1^Division of Toxicology, Wageningen University, Building 124, Stippeneng 4, 6708 WE Wageningen, The Netherlands.

^2^Aqaba international laboratories/BENHAYYAN, ASEZA, Aqaba 77110, Jordan

^3^Department of Biochemistry, Wageningen University, Dreijenlaan, 6703 HA Wageningen, The Netherlands

^4^Philadelphia University, Faculty of Pharmacy, P.O. Box 1 Amman 19392 Jordan

**Corresponding author:**

Amer J. Al-Malahmeh,

Division of Toxicology, Wageningen University

Tuinlaan 5, 6703 HE Wageningen, the Netherlands.

Tel.: 0031 317 483971

Fax: 0031 317 484931

E-mail: [amer.almalahmeh@wur.nl](mailto:amer.almalahmeh@wur.nl)


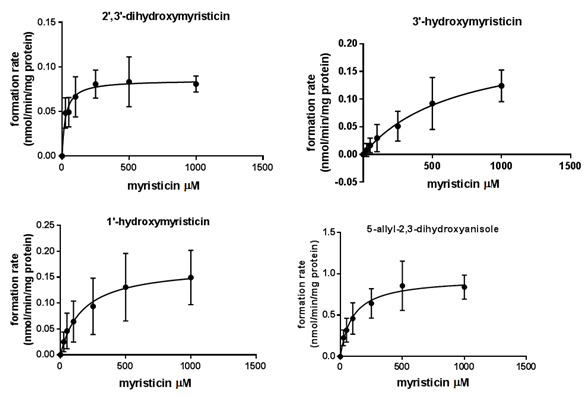


**Fig. S1** Concentration-dependent rate of myristicin metabolite formation in incubations with pooled human mixed gender liver microsomes. Curves for formation of four different phase I metabolites (2',3'-dihydroxymyristicin, 3'-hydroxymyristicin, 1'-hydroxymyristicin and 5-allyl-2,3-dihydroxyanisole) are presented.
